# Short-term effects of defoliation intensity on sugar remobilization and N fluxes in ryegrass

**DOI:** 10.1093/jxb/ery211

**Published:** 2018-06-21

**Authors:** Frédéric Meuriot, Annette Morvan-Bertrand, Nathalie Noiraud-Romy, Marie-Laure Decau, Abraham J Escobar-Gutiérrez, François Gastal, Marie-Pascale Prud’homme

**Affiliations:** 1 Université de Caen Normandie, INRA, UMR 950, Ecophysiologie Végétale, Agronomie et Nutritions NCS, Caen, France; 2 INRA, URP3F, Lusignan, France; 3 INRA, UR4, URP3F, Lusignan, France

**Keywords:** Defoliation, fructans, *Lolium perenne* L, nitrogen, remobilization, sucrose, uptake

## Abstract

In grassland plant communities, the ability of individual plants to regrow after defoliation is of crucial importance since it allows the restoration of active photosynthesis and plant growth. The aim of this study was to evaluate the effects of increasing defoliation intensity (0, 25, 65, 84, and 100% of removed leaf area) on sugar remobilization and N uptake, remobilization, and allocation in roots, adult leaves, and growing leaves of ryegrass over 2 days, using a ^15^N tracer technique. Increasing defoliation intensity decreased plant N uptake in a correlative way and increased plant N remobilization, but independently. The relative contribution of N stored before defoliation to leaf growth increased when defoliation intensity was severe. In most conditions, root N reserves also contributed to leaf regrowth, but much less than adult leaves and irrespective of defoliation intensity. A threshold of defoliation intensity (65% leaf area removal) was identified below which C (glucose, fructose, sucrose, fructans), and N (amino acids, soluble proteins) storage compounds were not recruited for regrowth. By contrast, nitrate content increased in elongating leaf bases above this threshold. Wounding associated with defoliation is thus not the predominant signal that triggers storage remobilization and controls the priority of resource allocation to leaf meristems. A framework integrating the sequential events leading to the refoliation of grasses is proposed on the basis of current knowledge and on the findings of the present work.

## Teaser

N uptake is positively correlated with photosynthetic capacity in ryegrass. The N sink strength of leaf meristems increased in response to defoliation. C and N reserves were remobilized only under severe defoliation.

## Introduction

In grassland plant communities, the ability of individual plants to regrow after defoliation is of crucial importance because it allows the restoration of active photosynthesis and plant growth. Ryegrass (*Lolium perenne* L.) is one of the predominant species in temperate grasslands because of its rapid establishment, tolerance to defoliation, high digestibility, and high herbage production. Four factors have been identified as the main components affecting regrowth. These are the leaf area remaining after defoliation through its remaining photosynthetic capacity (Parsons *et al.*, 1983; Lestienne *et al.*, 2006); stored carbohydrates (Volenec, 1986; Donaghy and Fulkerson, 1997; Morvan-Bertrand *et al.*, 1999*a*); stored nitrogen (N) (Ourry *et al.*, 1989; Volenec *et al.*, 1996; Schnyder and de Visser, 1999); and the number, status, and position of leaf meristems (Chapman and Lemaire, 1993; Gastal *et al.*, 2010; Gastal and Lemaire 2015). The relative importance of these factors varies with plant species and environmental conditions, which partly depend on grassland management. In the vegetative stage, the most important parameters are height (i.e. intensity) and frequency of defoliation (Parsons *et al.*, 1988; Thornton and Millard, 1997; Tunon *et al.*, 2013), together with N fertilization (Volenec and Nelson, 1983; Louahlia *et al.*, 1999, 2008).

When defoliation is severe and involves the removal of all leaf blades, photosynthesis decreases drastically or even ceases (Davidson and Milthorpe, 1966; Parsons *et al.*, 1983; Richards, 1993). Carbon (C) supply to leaf meristems then transiently depends on C reserves. In *L. perenne*, ^13^C labelling of pre-defoliation or post-defoliation C indicates that before the third day of regrowth leaf growth is mostly dependent on C reserves, and thereafter it mainly relies on current photoassimilates (de Visser *et al.*, 1997; Morvan-Bertrand *et al.*, 1999*b*). In most cool-season grasses, fructans (fructose polymers derived from sucrose) represent the main storage carbohydrates. They play an important role in buffering imbalances between photosynthetic C supply and C demand for growth, regrowth, and development (Volenec, 1986; Housley and Volenec, 1988; Morvan-Bertrand *et al.*, 2001; Amiard *et al.*, 2003). The leaf tissues remaining below the defoliation height, referred to as stubbles, are a heterogeneous compartment that includes fully expanded leaf sheaths and the basal parts of growing leaves. Leaf sheaths are generally considered to be the major C storage site in C3 grasses because they can accumulate up to 70% of the fructans stored in the vegetative parts. Elongating leaf bases also act as strong sinks for imported C assimilates, which are used not only to fuel the meristem for growth and respiration but also to synthesize substantial amounts of fructans (Allard and Nelson, 1991). Besides their storage role, fructans are subjected to continuous turnover (Pollock *et al.*, 2003), which may allow a fast net mobilization when needed. Remobilization of C occurs through fructan hydrolysis (Prud’homme *et al.*, 2007), sucrose synthesis from fructan-derived fructose, and increased sucrose transport activity (Berthier *et al.*, 2009). Recovery following defoliation is also related to the availability of N reserves previously accumulated within the remaining tissues, including leaf sheaths and elongating leaf bases (Gastal and Nelson, 1994). Indeed, N uptake and assimilation decline rapidly after defoliation (Clement *et al.*, 1978; Jarvis and Macduff, 1989; Ourry *et al.*, 1989; Thornton and Millard, 1997), together with a reduction or cessation in root growth and root respiration (Davidson and Milthorpe, 1966; Ryle and Powell, 1975; Ourry *et al.*, 1988). Remobilization of N from roots and stubble proteins occurs through proteolysis and the release of amino acids for incorporation into new growing tissues (Ourry *et al.*, 1989; Lefevre *et al.*, 1991; Schnyder and de Visser, 1999). Several studies on different plant species have demonstrated that carbohydrates and amino acids are involved in the regulation of nitrate uptake and in the coordination of nitrate uptake with light and photosynthesis (Muller and Touraine, 1992; Delhon *et al.*, 1996; Thornton, 2004; Louahlia *et al.*, 2008). Treatments that increase the intracellular concentration of amino acids in roots down-regulate the net uptake of N (Lee *et al.*, 1992; Muller and Touraine, 1992; Thornton, 2004; Louahlia *et al.*, 2008). Nitrate uptake is also under diurnal regulation, with net uptake being lower in darkness than in daylight (Thornton and Macduff, 2002; Lejay *et al.*, 2003). The external application of sucrose, glucose, and fructose via the nutrient solution at the beginning of the dark period prevents the decline of nitrate uptake and of corresponding expression of transporters in *Arabidopsis thaliana* (Lejay *et al.*, 1999, 2003). An external supply of sucrose in the nutrient solution of *L. perenne* at the time of defoliation also counteracts the decline of nitrate uptake in defoliated plants, suggesting that the down-regulation of nitrate uptake following defoliation might be effected through a shortage of carbohydrates in roots (Louahlia *et al.*, 2008). Therefore, modulating the intensity of defoliation through the removal of more or less leaf area should lead to differences in the N uptake rate and offer the opportunity to better study the interactions between C and N sources for regrowth, whether coming from storage remobilization or from current uptake and photoassimilation.

In the vegetative stage, grasses are well adapted to defoliation for two reasons. Their leaf meristematic zone is located close to ground level, and is thus protected from mowing and animal grazing, and their C and N storage compounds are mainly accumulated in shoot bases and therefore in remaining tissues; both of these factors allow the plant to rapidly regenerate new leaves after defoliation. Nevertheless, defoliation height may affect not only the remaining leaf area, but also the integrity of the leaf growth zone and the amounts of stored carbohydrates and N compounds (Lestienne *et al.*, 2006). Defoliation frequency mainly affects the replenishment of N and C storage (Thornton and Millard, 1997; Lasseur *et al.*, 2007; Lee *et al.*, 2010). Plant responses to defoliation height and frequency vary depending upon the form of growth, morphology, genetic capabilities, and physiology (Chapman and Lemaire, 1993; Lasseur *et al.*, 2007; Gastal and Lemaire 2015). The molecular signals down-regulating mineral N uptake and up-regulating N and C remobilization are, however, still largely unknown. Moreover, the relative patterns of N uptake and N and C remobilization are affected by the plant’s N status before defoliation (Louahlia *et al.*, 1999, 2008), the remaining leaf area (Lestienne *et al.*, 2006), and the atmospheric CO_2_ concentration (Thornton *et al.*, 2002); these factors exert their effects through complex interactions, making causal events difficult to establish. Finally, an external supply of fructose to the leaf sheaths of defoliated plants prevented fructans from being hydrolyzed (Amiard *et al.*, 2003). This finding suggests that fructan hydrolysis depends on the amount of C circulating in phloem and that a threshold of leaf area removal below which fructans are not used for regrowth might exist.

In order to gain a better understanding of the interactions between N and C metabolisms and therefore propose a better scheme of post-defoliation physiological events during the regrowth of *L. perenne*, the present study aimed to evaluate the short-term effects (2 d) of defoliation intensity on C remobilization, N uptake, N remobilization, and N allocation in roots, adult leaves, and growing leaves of *L. perenne*. Previous experiments on severely defoliated plants highlighted that during this critical period (2 d) leaf regrowth depends on N and C stored prior to defoliation (Morvan-Bertrand *et al.*, 1999*b*, de Visser *et al.*, 1997), but did not take into account the effect of defoliation intensity itself. Defoliation intensity was assessed by Lestienne *et al.* (2006) over a 7 d period but without taking into account the mobilization of C reserves. Therefore, in the present study, the defoliation intensity treatment consisted of the removal of an increasing proportion of leaf area (0, 25, 65, 84, and 100%, the latter representing plants that were totally defoliated). Analysis of N and C key storage compounds, including amino acids, proteins, and fructans, was combined with a ^15^N tracer technique to quantify N uptake, remobilization, and allocation over the 2 d period following defoliation. The hypotheses of this study were (i) that increasing defoliation intensity modulates C remobilization and N uptake, which in turn modulate N remobilization for fuelling primarily the growth of new leaves and then the regrowth of roots, and (ii) that a threshold defoliation intensity exists below which C and N storage compounds are not drawn upon for regrowth.

## Materials and methods

### Growth of plant material

**Fig. 1.**
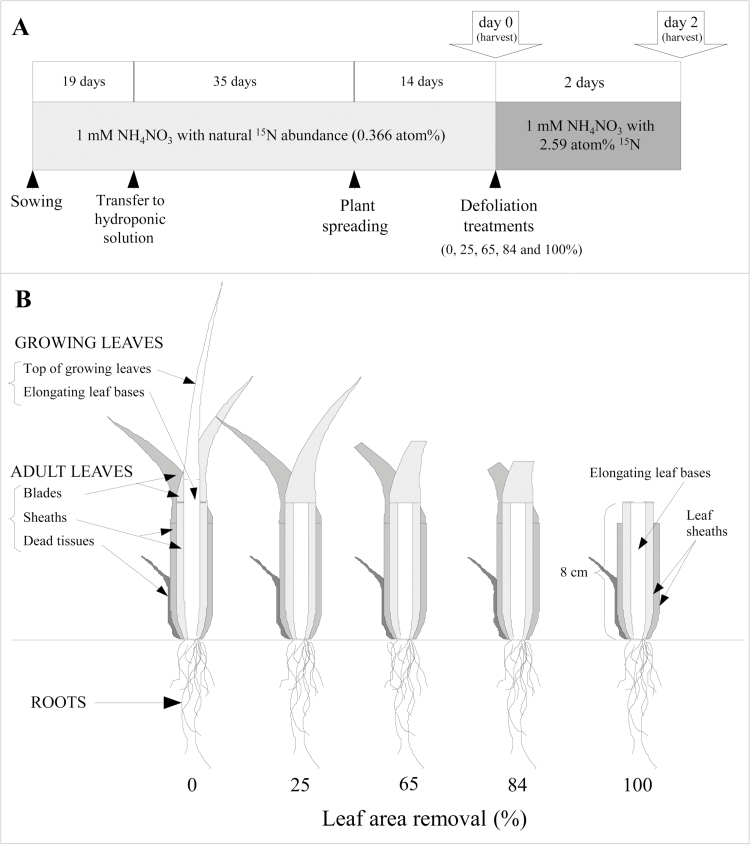
(A) Schematic representation of growing conditions and ^15^N labelling. (B) Schematic representation of plants reduced to a rooted tiller with three leaves and treatments applied at day 0: 0 (undefoliated plants), 25, 65, 84, and 100% (totally defoliated plants) leaf area removal. The different parts of leaves that were growing at the time of defoliation and continued to grow after defoliation, and adult leaves that were left after defoliation, are labelled.

Seeds of *Lolium perenne* L. cv. Vigor were germinated on filter paper moistened with deionized water within closed Petri dishes placed in a growth chamber (Froid et Mesures, Beaucouzé, France). The dishes were kept at 24 °C in the dark for 3 d and then transferred to light in the same growth chamber. Nineteen days after sowing, seedlings were transferred on to hydroponic solution aerated with 1 litre air h^−1^ and grown for 35 d at a density of 57 plants per m^2^ in a controlled environment growth chamber at a constant temperature of 18 °C and 70% relative humidity with a 14 h photoperiod of 289 µmol m^−2^ s^−1^ photosynthetic photon flux density (PPFD) at canopy height provided by a balanced mixture of metal halide and high-pressure sodium bulbs (HQI, 400 W, Osram, Munich, Germany). After 35 d, plants were sampled in order to maximize plant homogeneity and allow an accurate calculation of N remobilization and partitioning. Sampled plants were spaced at a density of 28.5 plants per m^2^ and grown for 14 d at a constant temperature of 18 °C and 75% relative humidity with a 14 h photoperiod of 427 µmol m^−2^ s^−1^ PPFD (Fig. 1). The nutrient solution contained 1.00 mM NH_4_NO_3_, 0.40 mM KH_2_PO_4_, 0.25 mM K_2_HPO_4_, 0.25 mM K_2_SO_4_, 1.50 mM CaCl_2_·2H_2_O, 0.50 mM MgSO_4_·7 H_2_O, 0.05 mM NaCl, 0.03 mM FeSO_4_·H_2_O, 0.10 mM EDTA, 0.025 mM H_3_BO_3_, 0.0005 mM CuSO_4_·5 H_2_O, 0.002 mM MnSO_4_·H_2_O, 0.0005 mM Na_2_Mo_4_·2H_2_O, and 0.002 mM ZnSO_4_·7H_2_O.

### Defoliation treatment and ^15^N labelling

The defoliation treatment consisted of an increasing defoliation intensity, in which different percentages of leaf area were removed (0, 25, 65, 84, and 100%) (Fig. 1). Plants with 100% leaf area removal were those where the adult leaf blades and the top of growing leaves were removed. The treatment corresponding to 25% leaf area removal was achieved by removing only the top of growing leaves (down to 8 cm from the shoot base) emerging from the tube formed by the leaf sheaths. The treatments corresponding to 65, 84, and 100% leaf area removal were achieved by removing the emerged part of the growing leaves (similarly, down to 8 cm from the shoot base) and by additionally cutting the distal part of the adult blades in order to obtain a chosen residual length of blades of 10 cm (for 65%), 4 cm (for 84%), or 0 cm (for 100%). Therefore, the leaf area removed varied as indicated but the entire leaf growth zone, located within the basal 8 cm of the growing leaf, was left intact in all treatments. All removed clippings were saved in order to measure the removed leaf area, using a leaf area scanner (LI-3100C, LI-COR, Lincoln, NE, USA). A first sampling and harvest was conducted by treatment at day 0 on a first set of plants. On these plants, the remaining leaf area was also measured so that the percentage of removed leaf area for each treatment could be accurately calculated. Four plant replicates were harvested for each treatment.

Immediately after the defoliation treatments and day 0 sampling, the remaining plants were supplied with a nutrient solution identical to that previously supplied except that N was labelled with ^15^NH_4_^15^NO_3_ with a ^15^N abundance of 2.59 atom% (i.e. source ^15^N abundance). The plants were allowed to regrow for 2 d. A second set of plants was then sampled and harvested per treatment (day 2).

### Harvesting and analysis of plant material

At harvest, plants were carefully rinsed with deionized water and divided into roots and shoots. Each tiller was then dissected into adult and growing leaves. Adult leaves were dissected into blades, sheaths, and dead tissues. Growing leaves were dissected into the top of growing leaves, corresponding to the emerged part, and elongating leaf bases, corresponding to the base still enclosed in the sheath tube (Fig. 1).

After harvest, all samples were freeze-dried, weighed, and ground. Total N content and ^15^N abundance ([^15^N/(^14^N+^15^N)]×100) of the ground samples were measured using a continuous flow isotope mass spectrometer (Twenty-twenty, Europa Scientific Ltd, Crewe, UK) linked to a C/N analyser (Roboprep CN, Europa Scientific Ltd).

For soluble protein extraction, 50 mg (leaf sheaths) or 25 mg (elongating leaf bases) of freeze-dried plant tissue, ground to a fine powder, were placed in a 14 ml polypropylene round-bottom tube with 2 ml of 100 mM sodium phosphate buffer (pH 7.4) at 4 °C. The tube was mixed by vortex three times for 30 s. The sample was centrifuged at 3200 *g* for 10 min at 4 °C and the supernatant was reserved. A 2 ml volume of buffer was added to the remaining pellet and the sample was mixed and centrifuged again. The two supernatants were pooled and used for soluble protein analysis. Soluble proteins were analysed in the supernatant by the protein–dye binding method of Bradford (1976), using bovine serum albumin as a standard.

For extraction of water-soluble carbohydrates, amino acids, and nitrate, 50 mg (leaf sheaths) or 25 mg (elongating leaf bases) of freeze-dried plant tissue, ground to a fine powder, were placed in a 14 ml polypropylene round-bottom tube with 2 ml of 80% ethanol (v/v) with 0.5 g l^−1^ mannitol as an internal standard. The tube contents were mixed and incubated for 15 min at 80 °C. After ethanol extraction, the sample was centrifuged at 10000 *g* for 10 min. The supernatant was reserved and 2 ml of pure water was added to the pellet. The tube content was mixed and incubated for 15 min at 60 °C. After this first aqueous extraction, the sample was centrifuged at 10000 *g* for 10 min. The supernatant was reserved and pooled with the ethanol supernatant. The aqueous extraction was repeated once with the pellet. The three supernatants were finally pooled, evaporated to dryness under a vacuum, and the residue was dissolved in 0.5 ml pure water. For analysis of water-soluble carbohydrates, aliquots of soluble extract (100 µl) were passed through mini-columns (Mobicols, MoBITec, Göttingen, Germany) packed, from bottom to top, with 150 µl of Amberlite CG-400 II, formate-form (Fluka, Buchs, Switzerland), 80 µl of polyvinylpolypyrrolidone (Sigma-Aldrich, St. Louis, MO, USA), and 250 µl of Dowex 50W X8-400 H^+^-form (Sigma-Aldrich, St. Louis, MO, USA) to remove pigments and charged compounds. Fructans, sucrose, glucose, and fructose were analysed by HPLC on a cation exchange column (Sugar-PAK, 300 × 6.5 mm, Millipore Waters, Milford, MA, USA) eluted at 0.5 ml min^−1^ and 85 °C with 0.1 mM Ca-EDTA in water, using mannitol as an internal standard and a refractive index detector (Millipore Waters, Milford, MA, USA).

For analysis of amino acids, aliquots of soluble extract were used to determine total amino acids by spectrophotometry at 570 nm using a ninhydrin assay (80 mg SnCl_2_ in 50 ml of 200 mM citrate buffer, pH 5.0, mixed with 2 g ninhydrin in 50 ml dimethyl sulfoxide).

For nitrate analysis, aliquots of soluble extract were used to determine the nitrate concentration by ion chromatography using a DIONEX DX100 system (Dionex, Sunnyvale, CA, USA).

### Calculation of N uptake and allocation

‘Post-defoliation N’ was defined as N taken up between day 0 and day 2, and ‘pre-defoliation N’ as N deriving from N taken up before day 0 and remobilized between day 0 and day 2. The ^15^N labelling (^15^N abundance in %) of each compartment (roots, adult leaves, growing leaves) of plants allowed the determination of post-defoliation N content (equation 1) at day 2 in each compartment using the average ^15^N abundance of plant tissues sampled before labelling at day 0 (0.3742%):

$$\mathrm{Post}-\mathrm{defoliation Ncontent}=\frac{\left[({\mathrm{sample}}^{\mathrm{15}}\mathrm{N abundance}-\mathrm{pre}-{\mathrm{labelling}}^{\mathrm{15}}\mathrm{N abundance}\right)}{({\mathrm{source}}^{\mathrm{15}}\mathrm{N abundance}-\mathrm{pre}-{\mathrm{labelling}}^{\mathrm{15}}\mathrm{N abundance})]\times N\;content}$$ (1)

Subtraction of the post-defoliation N content from the total N content allowed determination of the pre-defoliation N content at day 2 in each compartment (equation 2):

Pre−defoliation Ncontent=Total N content−Post−defoliation Ncontent (2)

**Fig. 2.**
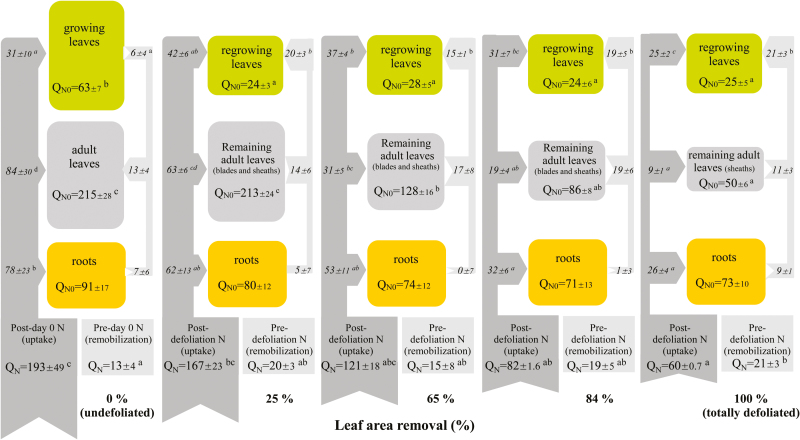
Effect of leaf area removal on N uptake, N remobilization, and N allocation between plant tissues over 2 d. In undefoliated plants, Q_N_ (mg N plant^−1^) represents N taken up (post-day 0 N) and N remobilized (pre-day 0 N) between day 0 and day 2. In defoliated plants (25–100%), Q_N_ (mg N plant^−1^) represents N taken up (post-defoliation N) and N remobilized (pre-defoliation N) between day 0 and day 2. Q_N0_ corresponds to the N amount of each tissue (mg N plant^−1^) on day 0 and determines the size of the box relative to the box representing undefoliated plants. The direction of arrows indicates the main direction of N flows entering or leaving the tissue (in % of Q_N_). The size of the arrows is quantitatively proportional to the N flow. Data are the means ±SE of four replicates. Different letters indicate significant differences between defoliation treatments (*P*<0.05; Tukey test).

Total N uptake by the plants (mg N plant^−1^) over the 2 d period of regrowth (Q_N_ in Fig. 2) was calculated using equation 3:

$$Total\;N\;uptake=\Sigma_{compartments}Post-defoliation\;Ncontent$$ (3)

Since the unlabelled N content of plants harvested on day 0 (total N content at day 0) was not statistically different from that of plants harvested on day 2 (data not shown), the plant can be considered as a closed system for unlabelled N from day 0 to day 2. Therefore, the allocation of unlabelled N between compartments observed in plants harvested on day 0 was applied to the unlabelled N content of plants harvested on day 2 to calculate their normalized unlabelled N allocation at day 0. It was then possible to calculate d2–d0 unlabelled N content differences for each compartment as described in Lestienne *et al.* (2006). The transfer of pre-defoliation N and post-defoliation N (in mg N plant^−1^) into or out of the different compartments was thus calculated according to equations 4 and 5 for each compartment:

$$Pre-defoliation\;Ntransfer_{\left(d2–d0\right)}=Pre-defoliation\;N_{d2}-Normalized\;unlabelled\;N_{d0}$$ (4)

$$Post-defoliation\;Ntransfer_{\left(d2–d0\right)}=Post-defoliation\;N_{d2}$$ (5)

An increase in unlabelled N content of any compartment from day 0 to day 2 was defined as an inward movement (i.e. influx, inlet) into that compartment (N import; sink compartment; transfer represented as an inward arrow in Fig. 2), while a loss of unlabelled N content from day 0 to day 2 was defined as an outward movement from that compartment (N export; source compartment; transfer represented as an outward arrow in Fig. 2).

Total pre-defoliation N remobilization by the plant (mg N plant^−1^) over the 2 d period of regrowth (Q_N_ in Fig. 2) was calculated using equation 6:

$$Total\;N\;remobilized=\Sigma_{source}{}_{compartments}Pre-defoliation\;Ncontent$$ (6)

The allocation of post-defoliation N in a compartment in percentage of the total post-defoliation N was calculated according to equation 7:

$$Post-defoliation\;N\;allocation=\frac{Post-defoliation\;N\;transfer(d2–d0)}{Total\;N\;uptake}\times 100\;\;$$ (7)

The allocation of pre-defoliation N was calculated as the relative amount of pre-defoliation N entering (inward) or leaving (outward) as a percentage of the total pre-defoliation N remobilized in the whole plant, according to equation 8:

$$Pre-defoliation\;N\;allocation=\frac{Pre-defoliation\;N\;transfer(d2–d0)}{Total\;N\;remobilized}\times 100\;\;$$ (8)

### Statistics

The resulting variation in measurements was expressed as the mean ±SE for *n*=4. Statistical analyses were performed using the software package R (R Development Core Team, 2007). After verifying that variance was homogenous (Bartlett test, 99%) and residuals were distributed normally (Shapiro–Wilk test, 99%), variables were compared between treatments by one-way ANOVA, followed by a Tukey test (95%). Correlation between N uptake and defoliation intensity was assessed by Pearson’s correlation coefficient.

## Results

### Plant dry mass, leaf area, and regrowth

*Lolium perenne* plants were progressively defoliated by the removal of 0, 25, 65, 84, and 100% of leaf area at the vegetative stage. On day 0, there were 94.3 ± 3.7 tillers per plant. As expected, the effect of removing an increasing percentage of shoot material on day 0 was still evident after 2 d of regrowth, with plants subjected to greater defoliation intensity on day 0 having a smaller relative growth rate, shoot and blade biomass, and leaf area on day 2 (Table 1). Relative growth rate was not very different between the treatments in which 0, 25, or 65% of leaf area was removed, but strongly decreased for the treatments in which 85% or 100% of leaf area was removed (Table 1). At the same time, the rate of leaf area production was barely affected by the intensity of defoliation. Indeed, the rate of leaf area production for undefoliated plants was only 4% higher (2.7 dm^2^ in 2 d) than for totally defoliated plants (2.59 dm^2^ in 2 d). By contrast, defoliation intensity had no impact on the biomass of sheaths, dead tissues, and roots, the production of growing leaves, and the number of tillers over the 2 d period (Table 1). In contrast to undefoliated plants, no new adult leaves were produced by totally defoliated plants during the 2 d period of regrowth.

**Table 1.** Biomass of the whole plant and plant tissues (root, shoot, adult and growing leaves), leaf area, and number of tillers per plant for the different treatments (0, 25, 65, 84 and 100% of leaf area removed) on day 0 and day 2

			Leaf area removed (%)					Effect
			0 (undefoliated)	25	65	84	100 (totally defoliated)	
Plant (g DM plant^−1^)	Whole plant	Day 0	13.4^b^ (1.84)	12.1^ab^ (0.81)	9.07^ab^ (1.71)	8.54^ab^ (1.92)	7.23^a^ (1.65)	*
		Day 2	16.3^b^ (4.14)	16.4^b^ (2.53)	11.4^ab^ (2.32)	9.41^ab^ (1.43)	7.76^a^ (0.74)	*
RGR (g g ^−1^ day^−1^)			0.111	0.179	0.129	0.051	0.036	
Plant (g DM plant^−1^)	Root	Day 0	4.16 (0.88)	3.98 (0.41)	3.49 (0.75)	3.41 (0.83)	3.61 (0.86)	ns
		Day 2	4.73 (0.87)	4.97 (1.08)	4.11 (0.97)	3.93 (0.69)	3.85 (0.66)	ns
	Shoot	Day 0	9.21^c^ (1.00)	8.07^bc^ (0.41)	5.58^ab^ (0.99)	5.13^ab^ (1.11)	3.62^a^ (0.87)	***
		Day 2	11.6^b^ (3.28)	11.4^b^ (1.66)	7.30^ab^ (1.49)	5.49^ab^ (0.76)	3.91^a^ (0.41)	*
Adult leaves (g DM plant^−1^)	Sheaths	Day 0	2.65 (0.47)	2.64 (0.15)	2.53 (0.49)	3.12 (0.74)	2.63 (0.65)	ns
		Day 2	3.40 (1.05)	4.03 (0.64)	3.05 (0.84)	2.93 (0.44)	2.28 (0.30)	ns
	Blades	Day 0	4.37^c^ (0.31)	4.37^c^ (0.25)	2.03^b^ (0.30)	1.05^a^ (0.21)	–	***
		Day 2	5.69^b^ (1.51)	5.09^b^ (0.73)	2.12^a^ (0.35)	0.87^a^ (0.09)	–	***
	Dead tissues	Day 0	0.47 (0.13)	0.28 (0.07)	0.29 (0.11)	0.26 (0.03)	0.23 (0.04)	ns
		Day 2	0.51 (0.17)	0.46 (0.10)	0.53 (0.14)	0.30 (0.14)	0.27 (0.06)	ns
Growing leaves (g DM plant^−1^)	Base	Day 0	0.82 (0.10)	0.78 (0.07)	0.73 (0.14)	0.70 (0.19)	0.74 (0.21)	ns
		Day 2	0.84 (0.26)	0.89 (0.14)	0.76 (0.11)	0.62 (0.09)	0.48 (0.06)	ns
	Top	Day 0	0.89 (0.06)	–	–	–	–	–
		Day 2	1.17^a^ (0.31)	0.92^ab^ (0.11)	0.84^a^ (0.09)	0.77^a^ (0.13)	0.78^a^ (0.09)	ns
Area (dm^2^ plant^−1^)		Day 0	18.1^b^ (1.62)	13.7^b^ (1.94)	6.21^a^ (0.65)	2.68^a^ (0.41)	–	**
		Day 2	20.8^c^ (3.95)	16.4^c^ (2.21)	8.68^b^ (0.95)	5.18^ab^ (0.70)	2.59^a^ (0.25)	***
Tillers (*n* plant^−1^)		Day 0	93 (7)	99 (7)	92 (10)	91 (12)	89 (10)	ns
		Day 2	107 (7)	111 (16)	113 (13)	96 (3)	101 (18)	ns

Values are means of four replicates with SE in parentheses. Treatment effect was tested by ANOVA: *P<0.05, ** *P*<0.01, ***P<0.001; ns, not significant. Significant differences between treatments are indicated by different letters (Tukey test). The relative growth rate (RGR) is calculated from the means of the whole plant DM at day 0 and day 2. DM, dry matter; –, no data available as the tissue does not exist.

### N uptake and allocation of pre- and post-defoliation N to plant compartments

The use of ^15^N allowed discrimination between the N acquired before (‘pre-defoliation N’, or remobilized N coming from N reserves) and after (‘post-defoliation N’, or N taken up between day 0 and day 2) defoliation, and to trace its allocation within the different plant compartments. In the present experiment, plants were initially cultivated in the presence of N at natural abundance, which was replaced by ^15^N-enriched N at day 0 when treated plants were defoliated (or not defoliated for the control plants). N flows and relative N allocation between plant parts during the 2 d of the experiment are presented in Fig. 2.

Undefoliated plants absorbed 193 mg N in 2 d. Increasing intensity of defoliation decreased, as expected, plant N uptake from 167 mg per plant in the least defoliated plants (25% leaf area removal) to 60 mg per plant in totally defoliated plants (100% leaf area removal). While plant N uptake was inversely correlated to increasing defoliation intensity (r=–0.993; *P*<0.001), plant N remobilization was not affected. As a consequence, the amount of remobilized N allocated to growing leaves was surprisingly similar in the various defoliation treatments. However, the proportion of remobilized N from adult leaves, relative to their N content on day 0 (Q_NO_), increased to a large extent (from 6 to 22%) with defoliation intensity.

The remaining adult leaves are composed of leaf sheaths and leaf blades in partially defoliated plants and only of leaf sheaths in totally defoliated plants. As expected, the sink strength of remaining adult leaves towards post-defoliation N compounds decreased when defoliation intensity increased. Indeed, post-defoliation N allocated to the remaining adult leaves declined from 63 mg to 9 mg per plant as defoliation intensity increased from 25% to 100% leaf area removal. The remaining adult leaves also became a source of pre-defoliation N for regrowing leaves, but the amount of N remobilized from remaining adult leaves (between 11 and 19 mg per plant) was not quantitatively linked to defoliation intensity.

As defoliation intensity increased, the amount of N taken up and subsequently allocated to regrowing leaves decreased from 42 mg to 25 mg per plant, but was relatively less affected than the amount of N taken up and subsequently allocated not only to the remaining adult leaves (from 63 mg to 9 mg per plant, as outlined above) but also to the roots (from 62 mg to 26 mg per plant). These latter values represent 40% on average of the N taken up by defoliated plants, independent of defoliation intensity. In contrast, the proportion of N taken up by defoliated plants and allocated to regrowing leaves increased with defoliation intensity (from 25% to 42%). Overall, these results lead to the conclusion that defoliation induced a relative increase of post-defoliation N allocation to regrowing leaves at the expense of roots and adult leaves. This effect is closely and positively linked to defoliation intensity.

Post-defoliation N remained the major source of N for regrowing leaves, but the relative contribution of pre-defoliation N increased with defoliation intensity when more than 65% of the leaf area was removed. In most conditions, root N reserves also contributed to the remobilization of N to regrowing leaves, but to a much smaller extent than the contribution of adult leaves, and independently of defoliation intensity.

### N metabolites and sugar contents

**Fig. 3.**
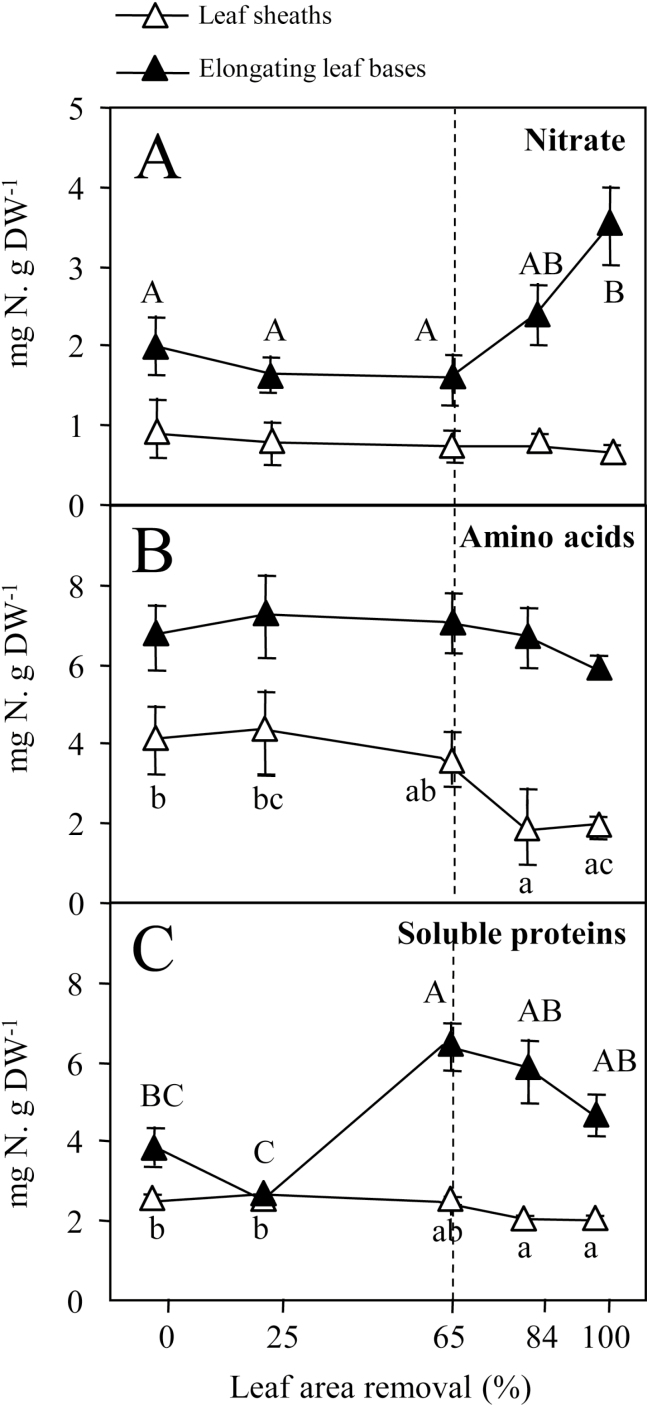
(A) Nitrate, (B) amino acid, and (C) soluble protein contents (mg N g^−1^ DW) in elongating leaf bases (closed symbols) and leaf sheaths (open symbols) of perennial ryegrass 2 d after the defoliation treatments. Values are the means of four replicates; error bars are SE when they extend beyond the symbol. Different letters indicate significant differences between defoliation treatments (*P*<0.05; Tukey test).

**Fig. 4.**
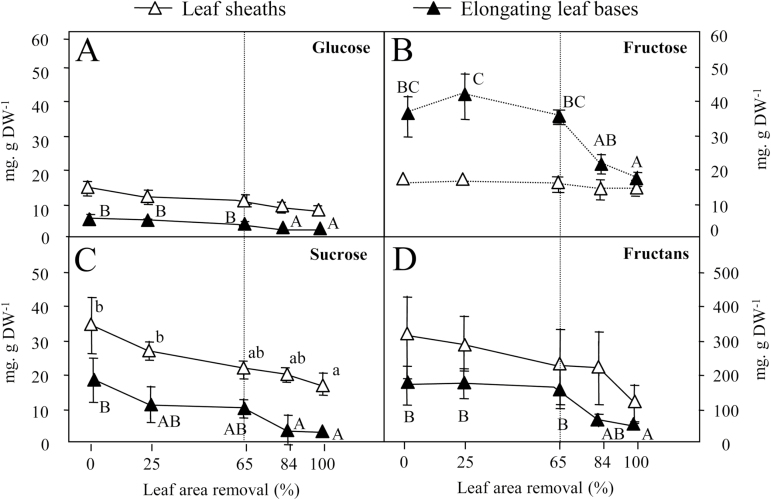
(A) Glucose, (B) fructose, (C) sucrose, and (D) fructan contents (mg g^−1^ DW) in elongating leaf bases (closed symbols) and leaf sheaths (open symbols) of perennial ryegrass 2 d after the defoliation treatments. Values are means of four replicates; error bars are SE when they extend beyond the symbol. Different letters indicate significant differences between defoliation treatments (*P*<0.05; Tukey test).

To gain better insight into the contribution of reserves to plant regrowth after defoliation, N metabolites (soluble proteins, total amino acids, and nitrate) (Fig. 3) and water-soluble carbohydrates (fructans, sucrose, glucose, and fructose) (Fig. 4) were quantified in elongating leaf bases and leaf sheaths. Elongating leaf bases correspond to the lower part of the growing leaves, enclosed in the whorl of leaf sheaths (Fig. 1), and include the leaf meristems. Each defoliation treatment removed the distal part of the elongating leaves (Fig. 1). In the vegetative stage, leaf sheaths can be considered as thoroughfares between leaf blades (where most photosynthesis occurs) and the rest of the plant. In undefoliated plants, the contents of nitrate, amino acids, and soluble proteins were greater in elongating leaf bases than in leaf sheaths (Fig. 3). Two days after defoliation, the nitrate content in leaf sheaths and the amino acid content in elongating leaf bases did not vary significantly between defoliation treatments (Fig. 3). By contrast, the nitrate content increased in elongating leaf bases, while the amino acid content declined in leaf sheaths, as did the soluble protein content in both tissues, when defoliation intensity exceeded 65% of leaf area removed (Fig. 3).

As the defoliation intensity increased, the sucrose content declined gradually in both leaf sheaths and elongating leaf bases (Fig. 4). The fructose and glucose contents did not vary significantly in leaf sheaths in response to defoliation. By contrast, they both decreased in elongating leaf bases in response to defoliation exceeding 65% of leaf area removed. The fructan content also declined in elongating leaf bases in response to defoliation intensity exceeding 65% of leaf area removed. In leaf sheaths, fructans declined only when plants were totally defoliated.

## Discussion

The objectives of this study were to assess (i) whether increasing defoliation intensity would modulate C remobilization and N uptake, which in turn would modulate N remobilization for fuelling first and foremost the growth of new leaves and then the regrowth of roots, and (ii) whether a threshold defoliation intensity might exist below which C and N storage compounds might not be drawn upon for regrowth.

### Effect of defoliation intensity on N uptake and partitioning

Defoliation intensity, in terms of the percentage of leaf area removed, affected total plant N uptake. Many studies have documented a negative effect of defoliation on root N absorption by grasses when compared with intact (i.e. undefoliated) plants (Richards, 1993). For example, N uptake per plant by *L. perenne* was reduced (Thornton and Millard, 1997) or even ceased over the following 4 d for totally defoliated plants (Ourry *et al.*, 1988). In addition, a single severe defoliation resulted in a large inhibition of NO_3_^−^ uptake by *L. perenne* compared with undefoliated plants for up to 8 d after defoliation (Jarvis and Macduff, 1989). Furthermore, when evaluating the impact of defoliation severity on *L. perenne*, plants cut to 4 cm height showed a greater reduction of N uptake compared with plants cut to 8 cm height (Thornton and Millard, 1996). This is probably the reason why in the present experiment cutting *L. perenne* plants to a height of 8 cm did not result in the cessation of N uptake.

Removal of more than 75% of the leaf area was required to reduce N uptake significantly when measured over a 7 d period (Lestienne *et al.*, 2006). In the present study, which focused on the first 2 d of regrowth, plant N uptake declined as defoliation intensity increased, with no threshold below which N uptake was not affected. Defoliation intensity thus has a greater impact on plant N uptake during the first few days after defoliation than later on.

The study of Louahlia *et al.* (2008) on *L. perenne* indicates that the shortage of carbohydrates in roots after defoliation could be regarded as a putative component of the regulatory mechanism for N uptake at the whole-plant level. Indeed, an external supply of sucrose in the nutrient solution at the time of defoliation counteracts the decline of nitrate uptake in defoliated plants, suggesting that down-regulation of nitrate uptake following defoliation might be effected through a shortage of sucrose in roots (Louahlia *et al.*, 2008). In the present study, the inverse relationship between remaining leaf area and down-regulation of N uptake after defoliation strongly suggests that N uptake is under the control of photosynthesis, probably through the transport of sucrose to the roots, given that sucrose represents the main form of C transport in *L. perenne* (Amiard *et al.*, 2004; Berthier *et al.*, 2009).

The shortage of carbohydrates in the roots of defoliated plants might also result from changes in the priority of C allocation in favour of leaf meristems at the expense of roots (Donaghy and Fulkerson, 1998). Priority is clearly given to the re-establishment of photosynthetic capacity through refoliation in order to prevent complete depletion of carbohydrates, which would be lethal for the plant. Our results also show that for the studies of defoliation responses, leaf area production might be a better parameter than relative growth rate to explain instantaneous plant performance. The present study also clearly demonstrates that with increasing defoliation intensity, post-defoliation and pre-defoliation N were preferentially allocated to growing leaves rather than to roots. Therefore, the sink strength of leaf meristems increases as a response to defoliation not only for C but also for N compounds. The mechanisms underlying this process are unknown. Defoliation gives rise to both wounding and removal of leaf area, which may trigger responses either independently or in concert. The fact that the sink strength of leaf meristems increased only above a threshold of defoliation intensity (65% of leaf area removed) does not suggest that there is exclusive control by wounding. The prioritization of resource allocation to the leaf meristems might result from the decline of sink strength in roots. Indeed, defoliation of grasses has been shown to result in decreased root biomass (Thornton and Millard, 1996). Following defoliation of *L. perenne* to a height of 4 cm, some root death was observed to occur over the following 13 d (Jarvis and Macduff, 1989). In the study of Lestienne *et al.* (2006), the final root biomass achieved on day 7 was shown to decrease as defoliation intensity was increased, and the amount of root N uptake over 7 d subsequently allocated to growing leaves was maintained at the expense of N allocation to roots and to adult leaves, which declined. However, according to the present study, 2 d following defoliation is not enough time to show significant changes in root biomass. Despite no observed changes in root biomass, sink strength increased in leaf meristems as an early response to defoliation. It can thus be concluded that the prioritization of C and N allocation to leaf meristems does not result from a decrease in root sink strength, but rather arises from a mechanism that occurs in the leaf meristems themselves in response to defoliation, linked to an imbalance between C supply and C demand for growth and development. This mechanism is still unknown (Bihmidine *et al.*, 2013).

### Effect of defoliation intensity on N and C remobilization

C and N reserves are remobilized to compensate for shoot removal and N uptake inhibition. This general response can be modulated by defoliation intensity.

Ourry *et al.* (1988) demonstrated that when ryegrass plants were totally defoliated, N was remobilized from roots and from stubble made up of leaf sheaths and elongating leaf bases, with stubble being preferentially depleted during the first 6 d of regrowth. Unexpectedly, in the present conditions, recycling of N compounds occurred from roots and growing leaves to adult leaves of undefoliated plants. However, it has been well established in other work on undefoliated grasses that remobilization of N occurs from adult leaves to growing leaves (Thornton *et al.*, 2002; Lehmeier *et al.*, 2010). The present results are probably due to the fact that some growing leaves became adult over the 2 d period of the experiment, so that growing leaves appear as a source compartment and adult leaves as a sink compartment for N reserves.

When integrated over 7 d of regrowth, measurement of N remobilization showed that more N was remobilized from roots to growing leaves as a response to defoliation (Lestienne *et al.*, 2006). This allowed the plants to compensate for the increased removal of shoot N reserves as defoliation intensity increased. In the legume *Medicago sativa* L., a similar increased contribution of roots in supplying remobilized N as defoliation intensity increased has been observed (Meuriot *et al.*, 2005). In the present experiment and when integrated over 2 d after defoliation, however, little N was remobilized from roots to growing leaves, regardless of defoliation intensity. Taken together, our results show that the pattern of N remobilization varies with defoliation intensity and with time during regrowth depending on the plant’s demand for N. By contrast with previous studies (Louahlia *et al.*, 1999), the present work shows that N remobilization did not increase proportionally to N uptake inhibition. Interestingly, N remobilization after defoliation can also occur independently of any down-regulation of N uptake in *Festuca rubra* (Thornton *et al.*, 2002). This suggests that N remobilization is not under the strict control of N uptake fluctuation through amino acid levels and/or cycling in the remaining vascular system, as discussed by Louahlia *et al.* (1999), but rather implies the presence of more complex and integrated factors at the whole-plant level. This suggests, in turn, either that there are distinct signal compounds to down-regulate uptake and up-regulate remobilization, or that there are different thresholds of the same signal for the induction of remobilization and down-regulation of N uptake, as discussed by Thornton *et al.* (2002).

Removal of more than 65% of the leaf area was required to significantly reduce both fructan content in elongating leaf bases after 2 d of regrowth and relative growth rate. Fructan content declined in leaf sheaths after 2 d only when plants were totally defoliated. Consequently, and in line with previous experiments (Amiard *et al.*, 2003), wounding associated with clipping is not the predominant signal that triggers fructan remobilization. Rather, fructan remobilization depends on defoliation intensity, and thus on the C assimilation capacity that remains after defoliation. We can hypothesize that when C assimilation is sufficient to sustain plant growth, fructans are preserved. When C assimilation is not sufficient, C stores are used to maintain leaf area production as a priority. Moreover, fructans are remobilized, only in elongating leaf bases if demand on C stores is low, or in both elongating leaf bases and leaf sheaths if demand on C stores for plant growth is high. Altogether, these results strongly suggest that fructan degradation is triggered by sugar depletion.

The size of the fructan pool is under the control of synthesis and breakdown enzymes, corresponding to fructosyltransferases (FT) and fructan exohydrolases (FEH), respectively. Sugar starvation due to defoliation induces fructan remobilization in the remaining leaf tissue during the first hours of regrowth, associated with a decrease of FT activity and an increase of FEH activity (Morvan-Bertrand *et al.*, 2001). Fructan degradation occurred concomitantly to sucrose decline in leaf sheaths and elongating leaf bases and to nitrate accumulation in elongating leaf bases. Modification of both nitrate and sucrose contents could explain the shift of fructan metabolism towards fructan hydrolysis. Indeed, besides its osmoregulating role, nitrate acts as a signal to repress fructan synthesis (Morcuende *et al.*, 2004), while sucrose is an inhibitor of fructan exohydrolase activity (Marx *et al.*, 1997; Lothier *et al.*, 2014) and a positive signal for FT expression (Müller *et al.*, 2000). Nitrate accumulation and sucrose content decline could lead to a decline in fructan content by repressing fructan synthesis and activating fructan hydrolysis by alleviating the sucrose inhibition of FEH. However, this does not account for the increase of FEH activity measured *in vitro* with no sucrose in the incubation medium, suggesting *de novo* FEH synthesis. *De novo* FEH synthesis has been reported in *Phleum pratense* in response to defoliation (Tamura *et al.*, 2011) but it has not yet been identified in *L. perenne* (Lothier *et al.*, 2007, 2014; Lee *et al.*, 2010).

Proteins serve as a store of N. However, regulation of proteolysis after defoliation has been poorly addressed in grasses, other than in species (*M. sativa*, *Trifolium repens*) that accumulate vegetative storage proteins (Goulas *et al.*, 2001; Noquet *et al.*, 2001). However, no vegetative storage proteins have been clearly identified in *L. perenne* (Louahlia *et al.*, 1999).

Nitrate content increased in elongating leaf bases when more than 65% of leaf area was removed, and this increase was positively correlated to the severity of the defoliation. Nitrate content increased concomitantly to the decrease in fructose, sucrose, and fructans. Nitrate accumulation, together with depletion of water-soluble carbohydrates, has previously been reported in the leaf growth zone of totally defoliated *L. perenne* plants over the following 2 d (Morvan-Bertrand *et al.*, 2001). This result, as well as those previously reported by Ourry *et al.* (1989), suggest that the increase of nitrate content in elongating leaf bases could compensate for the decline of soluble carbohydrates and therefore contribute to osmotic adjustment in expanding cells where water deposition is essential. However, nitrate accumulation is unexpected, since N uptake is inhibited at the same time. Nitrate accumulation might result from the decrease of nitrate reductase activity previously reported in the roots of totally defoliated plants (Boucaud and Bigot, 1989), together with the inability of elongating leaf bases to reduce nitrate (Gastal and Nelson, 1994). Nitrate reductase activity, which is known to be regulated by sucrose among other factors (Lillo, 2008), might thus be related here to the sucrose level, but this remains to be demonstrated.

### Integrative framework of events leading to the refoliation of grasses

The present study provides new insights into the interactions between C and N metabolisms occurring at the whole-plant level after defoliation and leading to subsequent leaf regrowth. It clearly demonstrates that (i) N uptake is positively correlated to photosynthetic capacity; (ii) C and N reserves are not necessarily remobilized, depending on photosynthetic capacity at the time of defoliation; (iii) there is no inverse relationship between N uptake and N remobilization; (iv) leaf meristem sink strength for N and C compounds increases in response to defoliation; and (v) priority is given to leaf regrowth at the expense of roots.

**Fig. 5.**
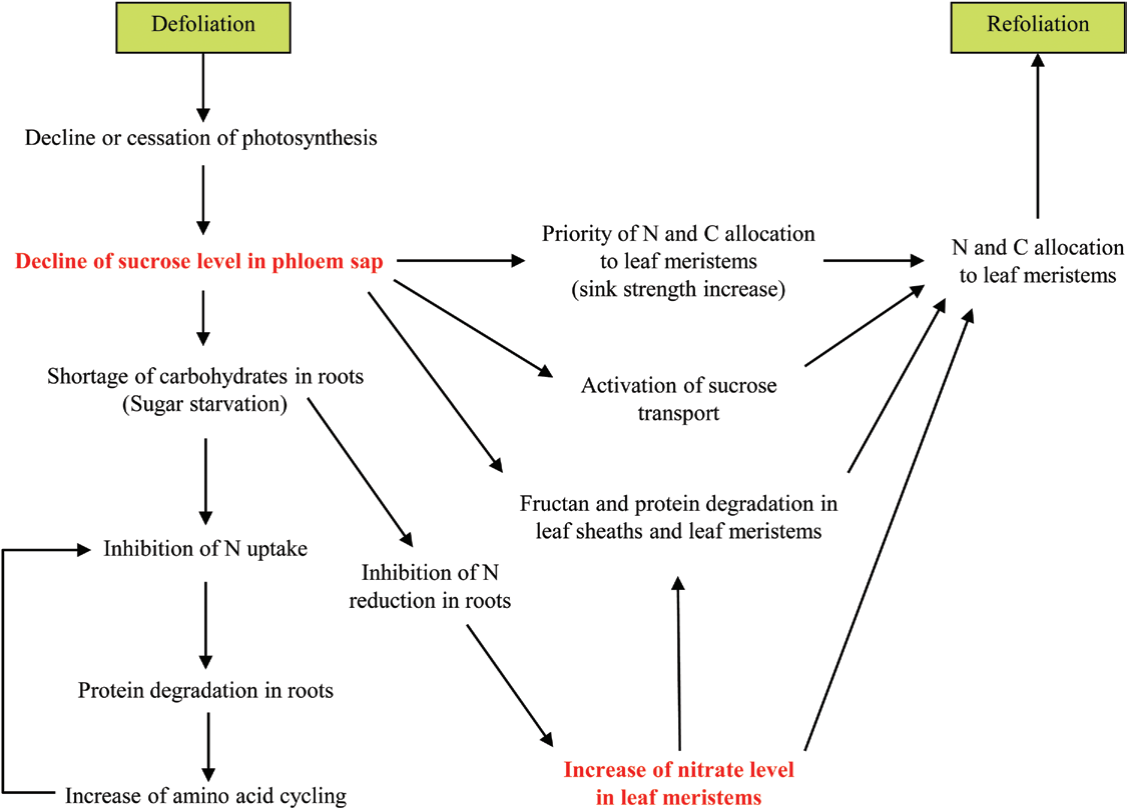
Integrative framework of the major events occurring after defoliation in grasses and leading to refoliation. Sucrose and nitrate signals are shown in bold.

In conclusion, we propose a framework that integrates the sequential events leading to the refoliation of grasses, based on current knowledge and our present findings (Fig. 5).

During the first hours of regrowth, the decrease or cessation of photosynthesis leads to a strong reduction in sucrose concentration in phloem sap (Amiard *et al.*, 2004). Together with the increase in the priority of C allocation to leaf meristems, this might result in a shortage of carbohydrates in the roots. The down-regulation of N uptake during the first hours of regrowth might be mediated first by sugar starvation and then by an increase in cycling of amino acids arising from proteolysis (Ourry *et al.*, 1989; Louahlia *et al.*, 2008). Sugar starvation might also inhibit N reduction in roots, leading to nitrate accumulation in leaf meristems through the xylem flux from roots (Thornton and Macduff, 2002) since there is no nitrate reduction in leaf meristems (Gastal and Nelson, 1994). The regulation of proteolysis and of fructan metabolism is still poorly understood. Sucrose and nitrate, whose levels decreased and increased, respectively, following defoliation, might act in concert as signals to trigger protein and fructan degradation (Marx *et al.*, 1997; Müller *et al.*, 2000; Morcuende *et al.*, 2004; Lothier *et al.*, 2010; Ould-Ahmed *et al.*, 2014). Other factors are involved in the control of protein and fructan metabolisms in relation to regrowth after defoliation, such as gibberellins (Morvan *et al.*, 1997; Cai *et al.*, 2016), abscisic acid (Yang *et al.*, 2004), and/or cytokinin (Wang *et al.*, 2013), which do not act as independent factors but are components of multiple signalling pathways.

More knowledge is necessary to determine the time frame of the sequential events and to better understand how the shifts between current assimilation and reserve mobilization are regulated, how N and C metabolisms are integrated at the molecular level, and the mechanisms by which priority of allocation is given to the leaf meristem in grasses to allow rapid leaf replacement after defoliation.
